# Autocrine/Paracrine Loop Between SCF^+^/c-Kit^+^ Mast Cells Promotes Cutaneous Melanoma Progression

**DOI:** 10.3389/fimmu.2022.794974

**Published:** 2022-01-24

**Authors:** Tiziana Annese, Roberto Tamma, Mariella Bozza, Alfredo Zito, Domenico Ribatti

**Affiliations:** ^1^ Department of Basic Medical Sciences, Neurosciences and Sensory Organs, University of Bari Medical School, Bari, Italy; ^2^ Istituto di Ricovero e Cura a Carattere Scientifico (IRCCS) Istituto Tumori Giovanni Paolo II, Bari, Italy

**Keywords:** angiogenesis, mast cells, melanoma, tumor progression, tumor microenevironment

## Abstract

c-Kit, or mast/stem cell growth factor receptor Kit, is a tyrosine kinase receptor structurally analogous to the colony-stimulating factor-1 (CSF-1) and platelet-derived growth factor (PDGF) CSF-1/PDGF receptor Tyr-subfamily. It binds the cytokine KITLG/SCF to regulate cell survival and proliferation, hematopoiesis, stem cell maintenance, gametogenesis, mast cell development, migration and function, and it plays an essential role in melanogenesis. SCF and c-Kit are biologically active as membrane-bound and soluble forms. They can be expressed by tumor cells and cells of the microenvironment playing a crucial role in tumor development, progression, and relapses. To date, few investigations have concerned the role of SCF^+^/c-Kit^+^ mast cells in normal, premalignant, and malignant skin lesions that resemble steps of malignant melanoma progression. In this study, by immunolabeling reactions, we demonstrated that in melanoma lesions, SCF and c-Kit were expressed in mast cells and released by themselves, suggesting an autocrine/paracrine loop might be implicated in regulatory mechanisms of neoangiogenesis and tumor progression in human melanoma.

## Introduction

Stem cell factor (SCF) is a mast cell growth factor (1) involved in mast cell survival and migration (2). One of the main chemoattractant factors released by tumor cells is SCF (2–4). c-Kit (CD 117) is a member of class III transmembrane receptor tyrosine kinases (RTKs), linked to the platelet-derived growth factor (PDGF)/colony-stimulating factor-1 (CSF-1) subfamily (5), and acts as a natural receptor of SCF (6). c-Kit is expressed in mast cells and is involved in their growth and development (7). Mice lacking c-Kit or its ligand result in the absence of mast cells and exhibit several other kit-dependent phenotypic abnormalities (8, 9). Inhibition of the SCF/c-Kit axis inhibits the migration of mouse bone marrow-derived cultured mast cells to tumors in a transplanted tumor model in mice (2), and mutations in c-Kit have been associated with the development of gastrointestinal stromal tumors, various forms of mastocytosis, and mast cell leukaemia (10).

SCF exists as two alternatively spliced variants, the membrane-anchored protein and the soluble one differing in exon 6. Both isoforms are initially membrane-anchored and are composed of an extracellular domain, a transmembrane portion and an intracellular domain, which is not present in the membrane-anchored form of SCF (11). The isoform that retains the exon 6 is rapidly cleaved to generate the soluble protein (12–14). Both isoforms, with some differences, have a role in c-Kit activation.

c-Kit exists as four isoforms that differ for four amino acid residues of the extracellular domain (15). The full-length membrane-anchored one activates the intracellular signalling pathways in response to SCF binding, while the soluble one retains only the extracellular domain (16, 17).

SCF binding to the c-Kit extracellular domain induces its dimerization and follow the activation of its intrinsic tyrosine kinase activity that couples with a set of cytoplasmic signalling pathways including mitogen-activated protein kinase pathways (MAPK), fosfoinositide 3-kinase (PI3K) and Src family kinases, resulting in cell proliferation, migration and malignant transformation associated with different genetic diseases and cancers (18, 19).

Mast cells are numerous in tissues and organs exposed to the external environment, including the skin, the lung, and the gut, and are often located near to potential targets of their mediators, such as glands and blood vessels (20, 21). Mast cells are attracted in the tumor microenvironment and promote tumor angiogenesis and lymphangiogenesis by releasing vascular endothelial growth factor (VEGF), fibroblast growth factor-2 (FGF-2), and proteases (22). In addition, mast cells support tumor invasiveness by their immunosuppressive activity, releasing tumor necrosis factor-alpha (TNF-α), histamine, and interleukin-10 (IL-10) and suppressing T cells and natural killer (NK) that in turn induce immune tolerance mediated by regulatory T cells (Treg) (22). Tissue-derived mast cells from human skin secrete several angiogenic molecules, including VEGF, granulocyte-macrophage colony-stimulating factor (GM-CSF), interleukin-8 (IL-8) monocyte chemoattractant protein-1 (MCP-1), and require SCF for their secretion (23).

Given the complexity of the SCF/c-Kit pathway and the multiple cells and physiological/pathological conditions in which it can be activated, the complete understanding of the biological roles of SCF/c-Kit is still an issue that deserves a thorough investigation. In this study, we have evaluated the expression of SCF and c-Kit in mast cells located near to the blood vessels and skin glands, during the tumor progression of human melanoma and we have correlated their expression with microvascular density.

## Materials and Methods

### Patients and Tissue Collection

This retrospective monocentric study included formaldehyde fixed and paraffin-embedded (FFPE) skin biopsies from 35 normal, premalignant lesions and melanoma patients who underwent curative resection (**Table 1**). Full-thickness skin samples containing epidermis, dermis, and hypodermis were collected from the archive of the Unità Operativa Complessa di Anatomia Patologica, IRCCS, Istituto Tumori “Giovanni Paolo II”, Bari, Italy. The study was approved by the local institutional Ethics Committees and followed the Helsinki Declaration of 1964 and later versions. All patients agreed to participate in the study and signed written informed consent. Skin biopsies were divided into four histological subgroups, namely normal skin (NS; n=5), common nevi (CN; n=10), dysplastic nevi (DN; n=10), melanomas at different stages (M; n=10).

**Table 1:** Clinical and histological information on patients.

	Normal skin^a^			
Average age (year)	46			
Men/women	1/4			
	**Common nevi**			
Average age (year)	37			
Men/women	4/6			
	**Dysplastic nevi** ^b^			
Average age (year)	56			
Men/women	2/8			
	***Melanomas***			
Average age (year)	64			
Men/women	4/6			
* Subclassification*	*(n=4)*	*(n=2)*	*(n=2)*	*(n=2*
* AJCC stage*	*IIA*	*IB*	*IIB*	*IIIC*
* Breslow Depth*	*T3a (2-3mm)*	*T2a (2 mm)*	*T3b (4 mm)*	*T4a (5 mm)*
* Clark level*	*IV*	*IV*	*IV*	*IV*
* Ulceration*	*absent*	*absent*	*present (1/2)*	*absent*
* Growth Phase*	*vertical*	*radial and vertical*	*vertical*	*radial and vertical*
* Mitoses/mm^2^*	*2-3*	*4-5*	*18-20*	*8-9*
* Tumoral lymphocytic infiltration*	*present (2/4)*	*present*	*absent*	*present*
* Peritumoral lymphocytic infiltration*	*present*	*present*	*present*	*present*
* Vascular invasion*	*absent*	*present*	*absent*	*absent*
**Overall**	**35**			

a All normal skin sections were from patients who underwent surgery for suspect breast cancer.

b All dysplastic nevi showed severe atypia but negative margins. AJCC, American Joint Committee on Cancer.

### CD31, VEGFA, c-Kit, and SCF Immunolabelling

FFPE blocks were cooled on ice and serially sectioned using a standard microtome. Three µm thickness tissue sections were dewaxed by oven-dried at 60°C for 1 hour and then immersed two times in fresh xylene for 5 minutes. Before starting staining protocols, the slides were rehydrated in an alcohol scale, rinsed for 10 min in Tris-buffered saline solution (TBS), and heated in a solution of sodium citrate pH 6.0 (ref. S1700, Agilent Dako) at 98°C in a water bath for 30 min. After cooling at room temperature for 30 min, the slides were rinsed in distilled water two times and one time in TBS for 1 min each. Then, tissue sections were washed in TBS + 0.025 Triton X-100 (TBS-Tr), and the endogenous phosphatases and peroxidases were blocked in an enzyme-blocking (ref. S2003, Agilent Dako) for 10 min. Afterwards, the sections were incubated with primary antibodies rabbit polyclonal IgG to CD31 (diluted 1:60; ref. ab28364, Abcam), or rabbit polyclonal IgG to VEGFA (diluted 1:200; ref. AB-90010, Immunological Sciences) or mouse monoclonal IgG1 to c-Kit (diluted 1:500; ref. AMAB90901, ATLAS Antibodies), or rabbit polyclonal IgG to SCF (diluted 1:1000; ref. ab64677, Abcam) for 30 min at room temperature. The sections were rinsed in TBS-Tr, incubated with biotinylated polymer for 15 min (ref. K5005, Agilent Dako), streptavidin-AP for 15 min (ref. K5005, Agilent Dako), and finally, the immunodetection was performed with a red chromogen for 20 min at room temperature (ref. K5005, Agilent Dako). In the end, all the sections were washed in distillate water, counterstained with Gill’s hematoxylin (ref. GHS132-1L, Sigma-Aldrich), and mounted in Glycergel (ref. C0563, Agilent Dako). As negative controls for primary antibodies specificity and nonspecific binding of the secondary antibodies, primary antibodies were omitted, or isotype-specific immunoglobulins at the same protein concentration as the primary antibody replacing the primaries antibodies were added.

### Morphometric Analysis

Immunolabeled slides were scanned using the whole-slide scanning platform Aperio ScanScope CS (Leica Biosystems, Nussloch, Germany) at 40x magnification, stored as high-resolution digital images, and analysed using the Aperio Positive Pixel Count algorithm or the counter tool embedded in the ImageScope v.11.2.0.780 (Leica Biosystems). Morphometric analysis was performed on three randomly selected fields/biopsy at 20x or 40x magnification using the rectangle tools to determine the specific areas of interest.

The analyses were performed in the same section area for all the staining by transferring the annotated region of interest across semi-serial slides.

**Figure 1:**
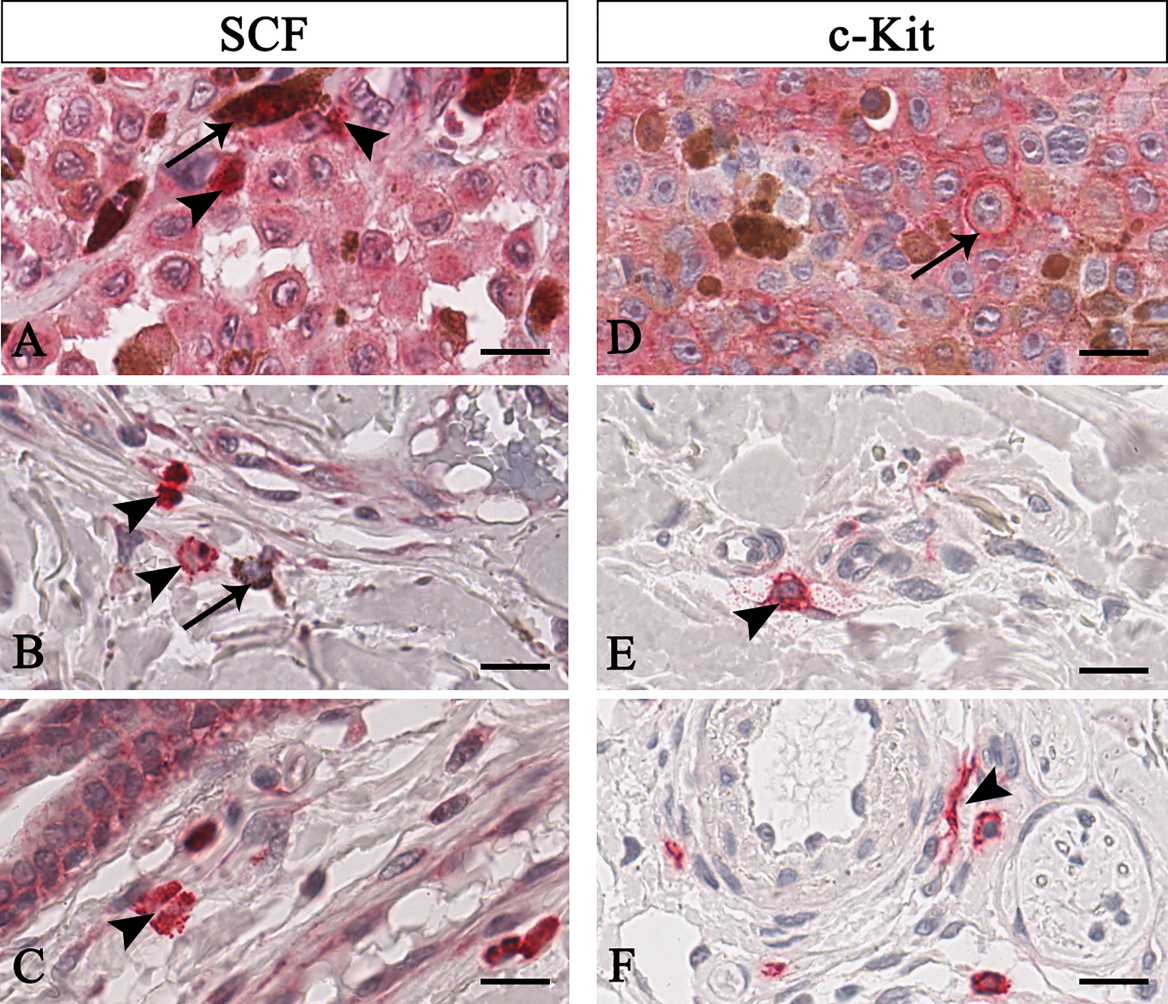
Representative immunohistochemistry of SCF **(A–C)** or c-Kit **(D–F)** in the melanomas tissue sections. Micrographs show pigmented tumor cells (**A**, arrow) or not-pigmented tumor cells but with nucleolated nuclei (**D**, arrow) labelled for both SCF or c-Kit. Instead, SCF^+^/c-Kit^+^ mast cells are rounded-shape and hyperchromatic cells with positive granules in the cytoplasm (**B**, arrowhead) or secreted (**A, C, E** arrowhead). Some mast cells are spindle-shaped, thus resembling fibroblasts (**F**, arrowhead). Scale bar: 60 μm.

The negative pen tool was used to draw negative free areas not to be analysed with the Aperio Positive Pixel Count algorithm in order to include data corresponding only to MCs in the analysis. In addition, for SCF^+^/c-Kit^+^ MCs counts, the other cell types were excluded based on morphology (see results paragraph and **Figure 1**).

### Statistical Analysis

Statistical analyses were performed using one-way ANOVA with Tukey’s multiple comparisons test, and proteins expression correlation was calculated assumed data sampled from no Gaussian distribution by Spearman nonparametric correlation analysis using the GraphPad Prism 6.01. Statistical significance was set at P ≤ 0.05, and results are given as mean ± SD.

## Results

### Mast Cells and Tumor Cells Express SCF and c-Kit in Normal, Premalignant, and Malignant Skin Lesions

To unequivocally quantify SCF/c-Kit of mast cells, we discriminate tumor cells based on their peculiar morphology. As shown in **Figure 1**, mast cells were identified as mononuclear cells containing many dense secretory granules covering the nucleus (**Figure 1** arrowhead), or cells with a dense red-positive plasma membrane signal as a crown (**Figure 1B**, arrowhead in the centre), or degranulated cells (**Figure 1A** arrowhead, see the soluble form of SCF; **Figure 1E**, see the soluble form of c-Kit), or elongated cells (**Figure 1F**). On the contrary, tumor cells appear to be pigmentated or not, faintly labelled for both SCF o c-Kit and with nucleolated nuclei (**Figures 1A, D** arrow). Based on these morphological profiles and employing the negative pen tool embedded in the ImageScope, we remove from the selected regions of interest the area of signals not related to mast cells.

### SCF^+^/c-Kit^+^ Mast Cells Distribution Around Microvessels in Normal, Premalignant, and Malignant Skin Lesions

In this study, we evaluate angiogenesis in normal, premalignant, and malignant skin lesions, which resemble steps of malignant melanoma progression, in relation to mast cells infiltrate around microvessels with the aim to identify a possible relation to mast cells distribution, SCF/c-Kit expression by mast cells, and microvessel formation.

**Figure 2:**
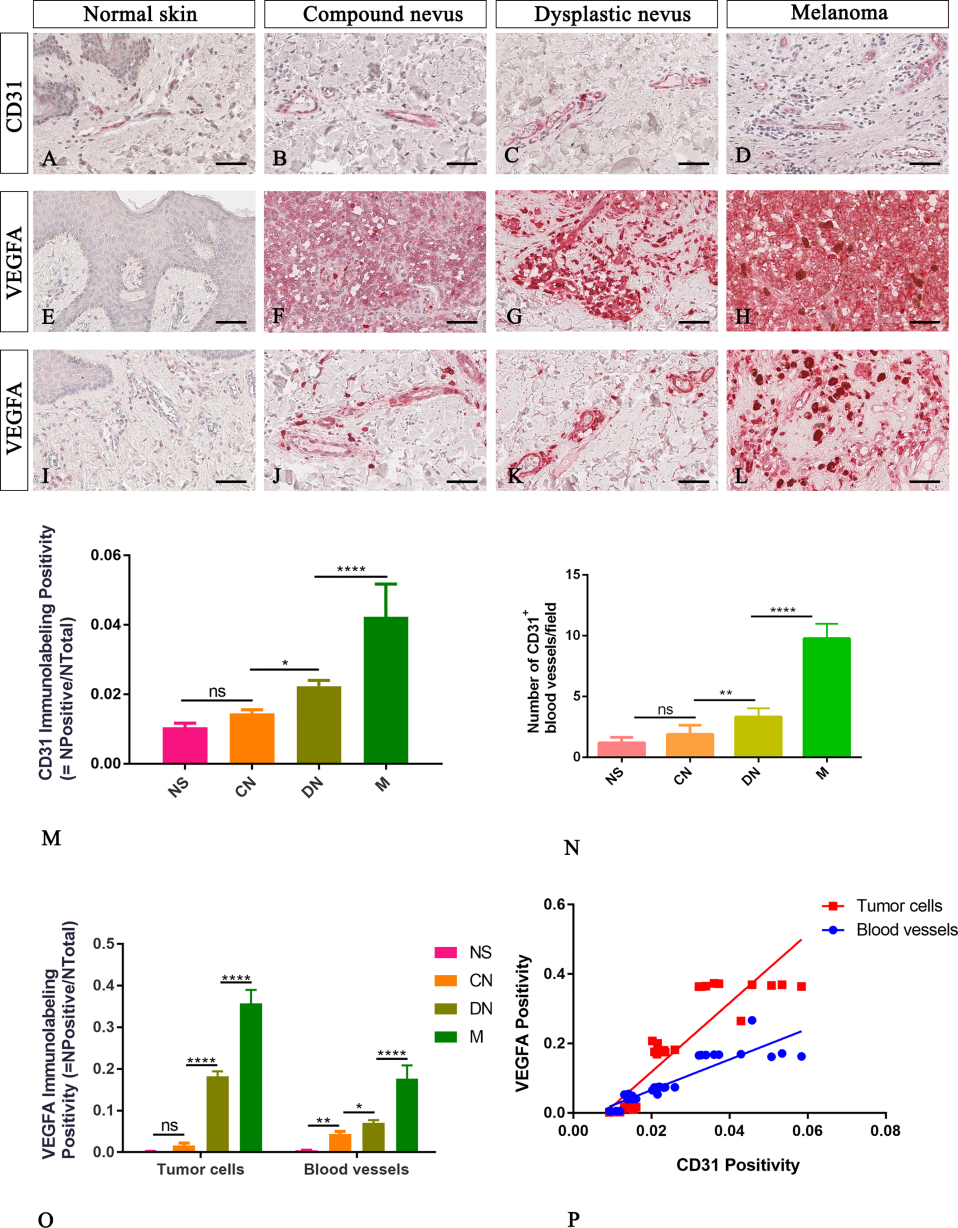
Representative immunohistochemistry of CD31 or VEGFA in the normal skin **(A, E, I)**, compound nevi **(B, F, J)**, dysplastic nevi **(C, G, K)**, and melanomas **(D, H, L)** tissue sections. Micrographs **(A–D)** and morphometric analysis **(M, N)** show a significative gradual increased microvascular density calculated as both percentages of CD31 immunolabeling positivity and the number of the CD31^+^ blood vessels in melanoma lesions compared to premalignant ones and normal skin. Micrographs **(E–L)** and morphometric analysis **(O)** show a significative gradual increased VEGFA expression in both cells of tumor mass **(E–H)** and surrounding blood vessels **(I–L)** in melanoma lesions compared to premalignant ones and normal skin. The lack of VEGFA expression is evident by the epidermis cells in the normal skin **(E)**, while a faint signal is present around the blood vessels **(I)**. Linear regression analysis shows positive relationships between VEGFA expression by tumor cells or by cells surrounding blood vessels and CD31 **(P)** [For VEGFA by tumor cells: y=9.929x-0.08029; R2 = 0.8095; p ≤ 0.0001. For VEGFA expression by cells surrounded blood vessels: y=4.407x-0.0225; R2 = 0.8117; p ≤ 0.0001]. Data are reported as means ± SD, and Tukey post-test was used to compare all groups after one-way ANOVA. Statistical significance: ns, not significative; *p ≤ 0.05; **p≤ 0.01; ****p ≤ 0.0001. Scale bar: 60 μm.

In agreement with the literature, our CD31 immunohistological staining showed a higher number of transversely or longitudinally cut blood vessels with an appreciable lumen in melanoma than normal skin, compound and dysplastic nevi (**Figures 2A–D**). The CD31 immunolabelling positivity and the number of CD31^+^ microvessels progressively increased from normal skin to melanoma (**Figures 2M, N**; **Table 2** for raw data), highlighting a significative involvement of angiogenesis in melanoma progression.

**Table 2:** Summary of the morphometric analysis performed to evaluate c-Kit^+^/SCF^+^ mast cells around blood vessels.

Analysis	EXPERIMENTAL GROUPS						
	NS	*vs*	CN	*vs*	DN	*vs*	M
CD31^+^ blood vessels immunolabeling positivity at 40x magnification	0.010496 ± 0.001164	ns	0.014543 ± 0.000963	*	0.022343 ± 0.001654	****	0.042335 ± 0.009406
Number of CD31^+^ blood vessels/field at 40x magnification	1 ± 0.5	ns	2 ± 1	**	3 ± 1	****	10 ± 1
VEGFA^+^ tumor cells immunolabeling positivity at 40x magnification	0.002165 ± 0.000317	ns	0.017022 ± 0.005223	****	0.182269 ± 0.011900	****	0.357288 ± 0.032636
VEGFA^+^ cells surrounding blood vessels immunolabeling positivity at 40x magnification	0.005185 ± 0.000186	**	0.044378 ± 0.005987	*	0.070713 ± 0.0006719	****	0.176649 ± 0.031862
CD31^+^ blood vessels immunolabeling positivity at 20x magnification	0.005016 ± 0.000720	ns	0.008073 ± 0.000556	**	0.014986 ± 0.000965	****	0.024751 ± 0.007599
SCF^+^ mast cells immunolabeling positivity at 20x magnification	0.018520 ± 0.001716	****	0.031347 ± 0.005586	****	0.060341 ± 0.004191	****	0.114667 ± 0.007065
c-Kit^+^ mast cells immunolabeling positivity at 20x magnification	0.008168 ± 0.000506	*	0.016015 ± 0.001027	***	0.024208 ± 0.001522	****	0.0444600 ± 0.005451
Number of SCF^+^ mast cells/field at 20x magnification	19 ± 3	*	25 ± 1	****	35 ± 4	****	49 ± 6
Number of c-Kit^+^ mast cells/field at 20x magnification	7 ± 1	***	17 ± 4	****	26 ± 5	****	44 ± 4

**Figure 3:**
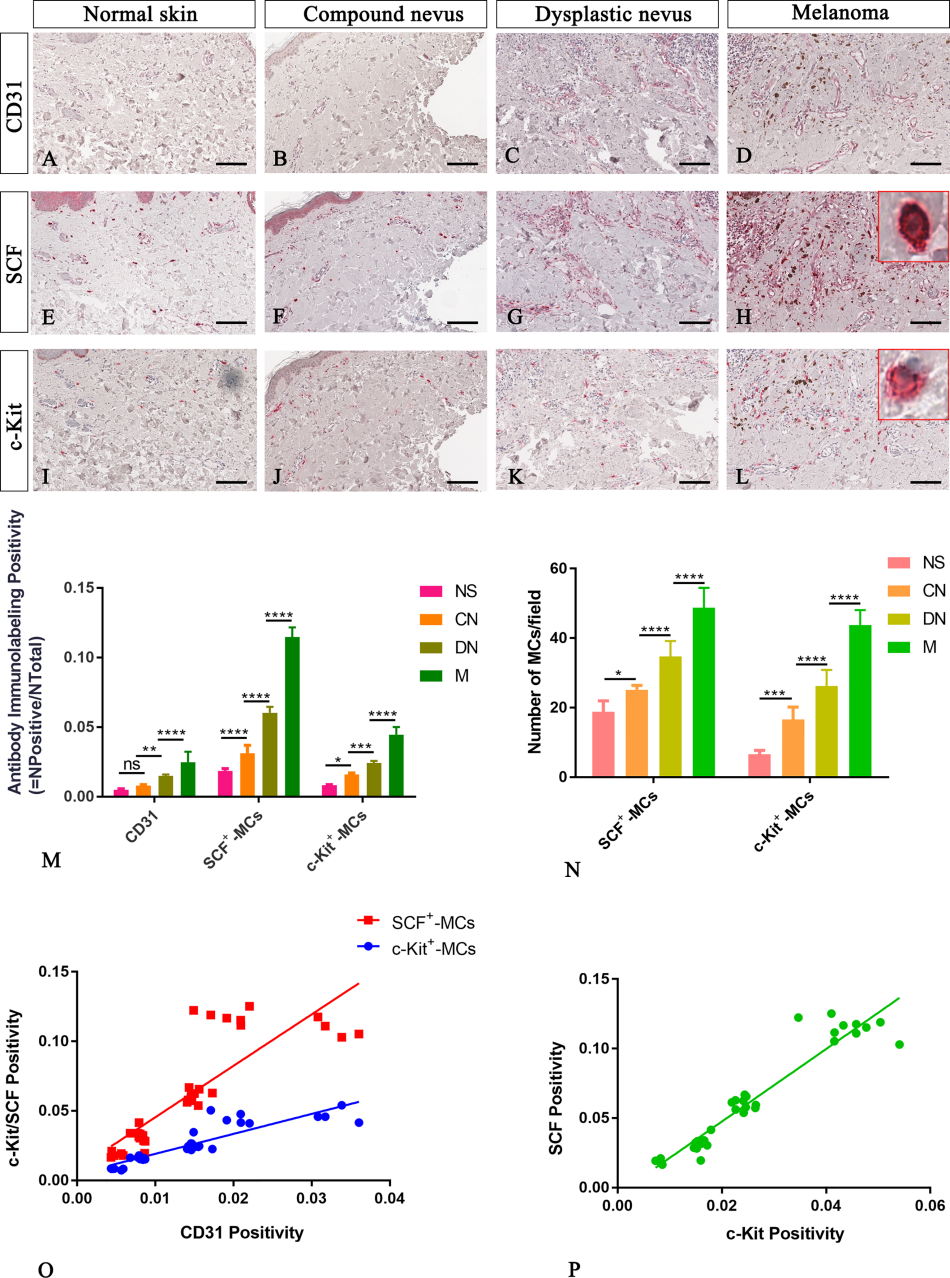
Representative immunohistochemistry of SCF or c-Kit on the same area of interest around CD31^+^ blood vessels in the normal skin **(A, E, I)**, compound nevi **(B, F, J)**, dysplastic nevi **(C, G, K)**, and melanomas **(D, H, L)** tissue sections. The micrographs show a significative gradual increased CD31, SCF, and c-Kit expression in malignant melanoma lesions compared to premalignant ones and normal skin **(A–L)**. The SCF- or c-Kit-positive mast cells appear mostly with a strong red-granulous membranous and moderate cytoplasmic staining in all the samples (see the inserts in the red rectangles; scale bar: 10 μm). The morphometric analysis performed on the same region of interest for all three markers confirmed an increased SCF^+^/c-Kit^+^ mast cell infiltrate around blood vessels in melanoma compared to normal skin, compound nevi, and dysplastic ones, both in percentages of immunolabeling positivity and number of positive cells **(M, N)**. Linear regression analysis shows positive relationships between SCF or c-Kit and CD31**(O)**, and between SCF and c-Kit **(P)** [**(O)** For SCF: y=3.698x+0.008438; R2 = 0.7048; p ≤ 0.0001. **(O)** For c-Kit: y=1.435x+0.004774; R2 = 0.792; p ≤ 0.0001. **(P)** y=2.61x-0.004682; R^2^ = 0.9123; p ≤ 0.0001]. Data are reported as means ± SD, and Tukey post-test was used to compare all groups after one-way ANOVA. Statistical significance: ns, not significative; *p ≤ 0.05; **p ≤ 0.01; ***p ≤ 0.001; ****p ≤ 0.0001. Scale bar: 200 μm.

**Figure 4:**
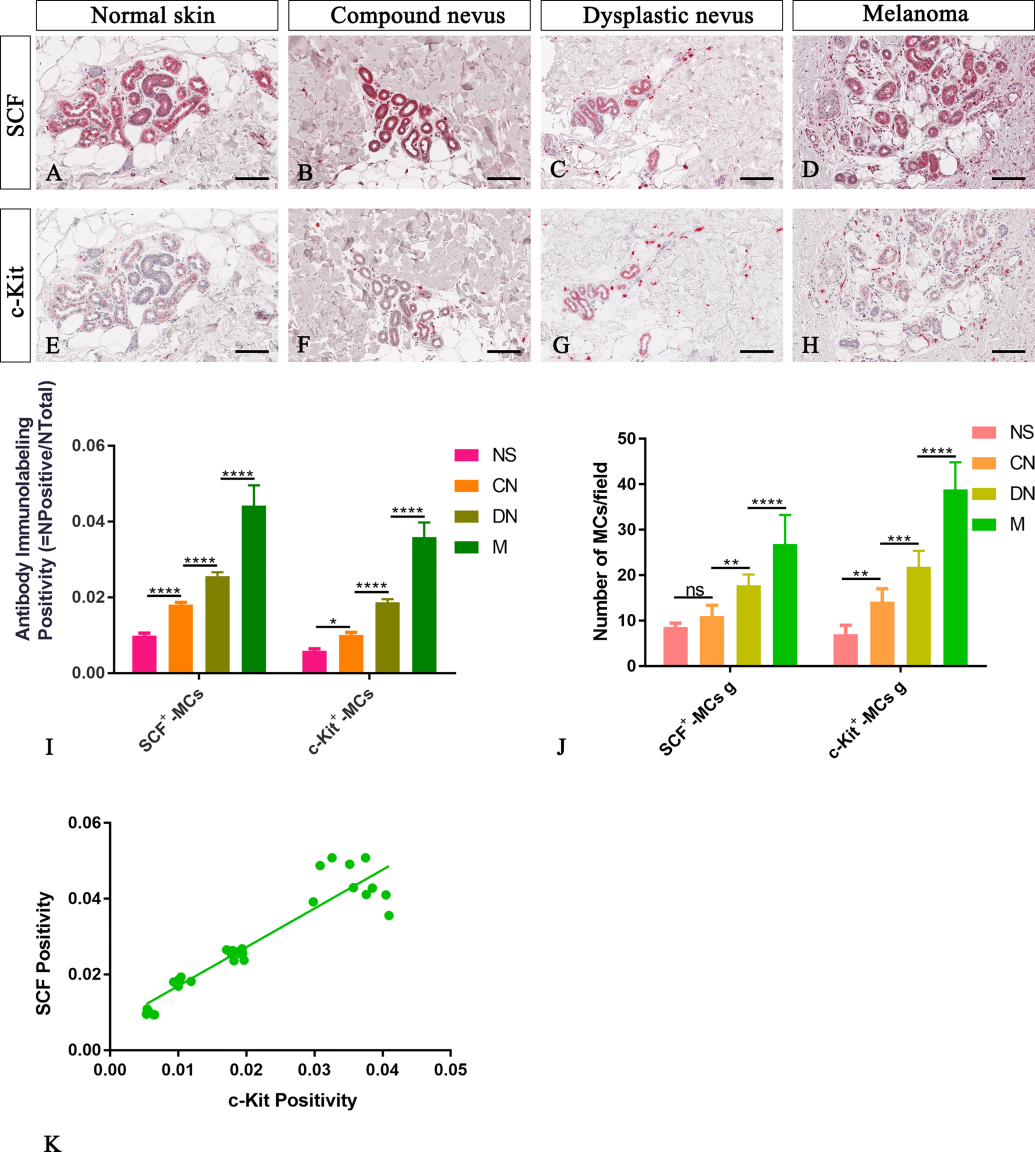
Representative immunohistochemistry of SCF or c-Kit on the same area of interest around glands in the normal skin **(A, E)**, compound nevi **(B, F)**, dysplastic nevi **(C, G)**, and melanomas **(D, H)** tissue sections. The micrographs show a significative gradual increased SCF and c-Kit expression in malignant melanoma lesions compared to premalignant ones and normal skin **(A–H)**. The morphometric analysis performed on the same region of interest for both markers confirmed an increased SCF^+^/c-Kit^+^ mast cell infiltrate around glands in melanoma compared to normal skin, compound nevi, and dysplastic nevi, both as percentages of immunolabeling positivity and as number of positive cells **(I, J)**. Linear regression analysis shows the positive relationships between **(K)** SCF and c-Kit [**(K)** y=1.023+0.006766; R^2^ = 0.8848; p ≤ 0.0001]. Data are reported as means ± SD, and Tukey post-test was used to compare all groups after one-way ANOVA. Statistical significance: ns, not significative; *p ≤ 0.05; **p ≤ 0.01; ***p ≤ 0.001; ****p ≤ 0.0001. Scale bar: 200 μm.

The morphometric values are expressed as mean ± SD (n= 5 for normal skin (NS) group and n=10 for the others) and have been utilised for the graphs in **Figures 2**–**4**. Tukey’s post-test was used to compare all groups after One-way ANOVA and Spearman for correlation (ns, not significative; *p ≤ 0.05; **p ≤ 0.01; ***p ≤ 0.001; ****p ≤ 0.0001).

Neo-angiogenesis mechanisms are related to the expression of angiogenic cytokines such as VEGFA. Its expression is usually absent in normal skin and nevi, but heterogeneous, ranging from negative to strongly positive, in melanomas (24, 25). To explore the angiogenic profile in our samples, we performed immunohistochemical analysis of VEGFA expression by tumor cells and cells surrounding the blood vessel. We found that VEGFA immunolabelling positivity progressively increased from normal skin to melanoma (**Figures 2E–L, O** and **Table 2** for raw data), and its expression amount, by both tumor cell or cell surrounding blood vessel, significative positive correlate with CD31 expression (**Figure 2P**). These results confirmed CD31 and VEGFA as good indicators of angiogenic changes in melanocytic lesions essential to multistep tumorigenesis.

Among cells of the microenvironment, mast cells take part in angiogenesis, tissue repair, and tissue remodelling that occurs in many tumor settings (26). Here, in normal, premalignant, and malignant skin lesions, we evaluate mast cells distribution in the connective tissue around CD31^+^ blood vessels as SCF^+^ or c-Kit^+^ cells with a masked nucleus, or cells with granules in the cytoplasm, or on the membrane, or outside the cells (as discerned in **Figure 1**). The analyses, conducted in the same region of interest on serial tissue sections for all the three markers, demonstrated an increased number of mast cells close to microvessels in melanoma lesions compared to premalignant ones and normal skin (**Figure 3**). The SCF or c-Kit immunolabelling positivity and the number of SCF^+^/c-Kit^+^ mast cells progressively increased from normal skin to melanoma (**Figures 3M, N**; **Table 2** for raw data). Moreover, correlation analysis of SCF^+^/c-Kit^+^ MCs and CD31^+^ microvessels and correlation analysis of SCF and c-Kit by linear regression showed significative positive correlations for both (**Figures 3O, P**).

### SCF^+^/c-Kit^+^ Mast Cells Distribution Around Cutaneous Glands in Normal, Premalignant, and Malignant Skin Lesions

Here, we examined SCF^+^/c-Kit^+^ mast cells distribution and location around the cutaneous gland in normal, premalignant, and malignant skin lesions by immunohistochemical staining because we observed that they not only surrounded the microvessels but also preferred to locate at extracellular matrix around the cutaneous glands. The analysis of immunolabeling reactions, conducted in the same region of interest on serial tissue sections for SCF and c-Kit, demonstrated an increased number of mast cells close to cutaneous glands in melanoma lesions than premalignant ones and normal skin (**Figure 4**). The number of SCF^+^/c-Kit^+^ mast cells progressively increased from normal skin to melanoma (**Figures 4I, J**; **Table 3** for raw data). Moreover, correlation analysis of SCF and c-Kit by linear regression showed significative positive correlations for both (**Figure 4K**). Overall, these results show a possible cross-talk between mast cells and gland epithelium mediated by the SCF/c-Kit pathway and involved in melanoma progression. As shown in **Figure 4** by SCF immunolabelling, the glandular epithelium cells can express SCF, which can bind the c-Kit express by the surrounded mast cells to sustain their proliferation, the pro-inflammatory microenvironment, therefore tumor maintenance and progression as tumor growth-promoting loop (27–29).

**Table 3:** Summary of the morphometric analysis performed to evaluate c-Kit^+^/SCF^+^ mast cells around glands.

Analysis	EXPERIMENTAL GROUPS						
	NS	*vs*	CN	*vs*	DN	*vs*	M
SCF^+^ mast cells immunolabeling positivity at 20x magnification	0.009901 ± 0.000678	****	0.018071 ± 0.000614	****	0.025568 ± 0.001080	****	0.044227 ± 0.005330
c-Kit^+^ mast cells immunolabeling positivity at 20x magnification	0.005852 ± 0.000549	*	0.010092 ± 0.000707	****	0.018666 ± 0.000847	****	0.03912 ± 0.003842
Number of SCF^+^ mast cells/field at 20x magnification	9 ± 1	ns	11 ± 2	**	18 ± 2	****	27 ± 6
Number of c-Kit^+^ mast cells/field at 20x magnification	7 ± 2	**	14 ± 3	***	22 ± 3	****	39 ± 6

The morphometric values are expressed as mean ± SD (n= 5 for normal skin (NS) group and n=10 for the others) and have been utilised for the graphs in **Figures 2**–**4**. Tukey’s post-test was used to compare all groups after One-way ANOVA and Spearman for correlation (ns, not significative; * p ≤ 0.05; ** p ≤ 0.01; ***p ≤ 0.001; ****p ≤ 0.0001).

## Discussion

In this study, we have demonstrated that in melanoma lesions, SCF and c-Kit are expressed in mast cells and released by themselves, suggesting an autocrine/paracrine loop might be implicated in regulatory mechanisms of neoangiogenesis and tumor progression in human melanoma.

SCF/c-Kit pathway activation regulates normal melanocytes, germ cells, and hematopoietic cells (included mast cells) proliferation and/or differentiation. SCF binding to the c-Kit monomer in its extracellular region induces dimerisation (30). The dimerised c-Kit acts as both enzyme and substrate through phosphorylation of its specific amino acid residues (pTyr) in the intracellular juxtamembrane region. Phosphorylated c-Kit tyrosine residues recruit signal trasduction protein-containing Src homology 2 (SH2) domains and pTyr-binding (PTB) domains (31). These downstream effector molecules have different biological effects: inhibit proliferation, or induce c-Kit degradation, cell proliferation, differentiation, survival, and migration (32). These effects will depend on total binding affinities and the number of recruitment sites (33). So this is why different cells show different phenotypes despite employing the same core sets of cell-signalling proteins. For instance, in the erythroleukemic K562 cell line and megakaryoblastic cell line MO7e, which expresses c-Kit and the phosphatases SHP-1/SHP-2 mRNA, under SCF stimuli and the inhibition of protein tyrosine phospahatase SHP-1/SHP-2 by the inhibitor NSC87877, increased cell proliferation, *via* PI3K-DAG-PKC signalling pathway was demonstrated through an increased c-Kit phosphorylation (32, 34).

The SCF/c-Kit pathway also plays a crucial role in tumor development, progression, and relapses (35). In epithelial ovarian cancer (EOC), the SCF/c-Kit autocrine/paracrine loop promotes cancer stem cell (CSC) survival, the leading cause of EOC initiation and relapse (36). In EOC, tumor cells (less amount) and c-Kit^+^ CSCs express the only membrane-anchored isoform of SCF, while tumor-associated macrophages (TAMs) and tumor-associated fibroblasts (TAFs) express even the secreted isoform (36).

Controversial is the literature concerning SCF/c-Kit transcript and protein level expression by tumor cells during melanoma progression. Some authors reported the loss of c-Kit expression during malignant transformation of melanocytes, but others, later in tumor progression, in invasive melanoma, compared to *in situ* lesions (37–40). Not only tumor cells but also cells of the microenvironment can express these molecules as secreted and/or membrane-bound isoforms for both (36, 41).

Human malignant melanoma is an aggressive and highly metastatic tumor with a poor prognosis and high drug resistance. Malignant melanoma progresses through different steps from common nevi, dysplastic nevi, *in situ* melanoma, radial growth phase melanoma, vertical growth phase melanoma, and metastatic melanoma (42). In parallel with tumor progression and dissemination, melanoma cells, in concert with microenvironment cells, orchestrate the angiogenic switch that favours tumor cell growth, extravasation and metastases (43, 44).

Tissue distribution and localisation of mast cells are intimately connected to their physiological and pathological functions, such as allergic immune reactions, innate and adaptive immunity, and fibrosis (45, 46). Tumor cells are surrounded by an infiltrate of inflammatory cells, such as lymphocytes, neutrophils, macrophages and mast cells. It is well known that mast cells accumulate at the periphery of melanomas and nevi, especially around the microvasculature, where have a pivotal role in neo-angiogenesis and metastasis mechanisms (47, 48). We have previously demonstrated a correlation between tumor vascularity and mast cell density and poor prognosis in melanoma (49), we have investigated the pattern of mast cells around the blood vessels in melanoma, and we have demonstrated that a higher number of mast cells can be observed in melanoma as compared with samples from common acquired nevi (50).

In this study, we have confirmed that CD31 immunolabelling positivity and the number of CD31^+^ microvessels in parallel to VEGFA expression by both tumor cells or cells surrounding blood vessels progressively increased from normal skin to melanoma, highlighting a significative involvement of angiogenesis in melanoma progression. Moreover, we have focused our attention on the role of mast cells positive to SCF/c-Kit in the initiation and progression of cutaneous melanoma. SCF is one of the principal growth factors responsible for the early development of CD34^+^/c-Kit^+^ mast cell progenitors and the development and function of melanocytes (51–54). SCF bind to the c-Kit tyrosine kinase receptor regulates mast cell development, survival, and function (35, 55). We have demonstrated that the number of SCF^+^/c-Kit^+^ mast cells progressively increased from normal skin to melanoma. In agreement with these results, also in pancreatic carcinoma, an increase in the number of chymase^+^/tryptase^+^ or SCF^+^/c-Kit^+^ mast cells in normal and tumor pancreatic tissue biopsies and cell lines was shown with a higher expression in the latter (56). Previous studies have reported the negative impact of SCF on c-Kit expression, demonstrating that a high SCF amount decreases the expression of c-Kit but does not affect the c-Kit phosphorylation (34, 57, 58). The immunohistochemistry on FFPE biopsy sections allows us to evaluate the biological phenomena in a context closer to the physiological one without overstating our data. Furthermore, it is very likely that in our study, the maintenance of a high expression of c-Kit is also attributable to other molecules and pathways active by other cells of the microenvironment. Zhang et al. (4) provided evidence that SCF released by tumor cells modulated tumor angiogenesis by regulating mast cells. These Authors used sense or anti-sense SCF cDNA to overexpress or deplete SCF expression in rat mammary tumor cells. Depletion of SCF significantly decreased mast cell infiltration and vascularisation, whereas the opposite effects were observed in SCF-overexpressing tumors.

Keratinocytes secrete SCF (4). SCF induces melanoblasts to differentiate into melanocytes and is mitogenic for melanocytes *in vivo* and *in vitro* (59). Injection of SCF into the human skin results in local accumulation of mast cells (60). c-Kit is a growth factor for melanocyte migration and proliferation and is massively involved in the tumorigenesis of cutaneous malignancies, being immunohistochemically densely expressed in dysplastic nevi and in melanoma. Melanocytes from metastatic melanoma lose the expression of c-Kit and an inverse correlation between c-Kit and tumor progression has been demonstrated in melanoma (61). In metastatic malignant melanoma, c-Kit staining was lower than in primary malignant melanoma (62).

In this context, we suggest that SCF^+^/c-Kit^+^ mast cells may be involved in the angiogenic response occurring during tumor progression in human melanoma. Mast cells are involved in an autocrine/paracrine loop that could stimulate the endothelial cell proliferation to sustain neo-angiogenesis, and the releasing of pro-inflammatory mediators and pro-angiogenic cytokines by themselves, which, in turn, sustain proliferation and activation of mast cells and tumor progression.

## Data Availability Statement

The raw data supporting the conclusions of this article will be made available by the authors, without undue reservation.

## Ethics Statement

The studies involving human participants were reviewed and approved by Istituto Tumori Giovanni Paolo II, Bari. The patients/participants provided their written informed consent to participate in this study.

## Author Contributions

TA and DR conceived, planned, and write the work. TA, RT, MB, and AZ performed the experimental work. All authors contributed to the article and approved the submitted version.

## Conflict of Interest

The authors declare that the research was conducted in the absence of any commercial or financial relationships that could be construed as a potential conflict of interest.

## Publisher’s Note

All claims expressed in this article are solely those of the authors and do not necessarily represent those of their affiliated organizations, or those of the publisher, the editors and the reviewers. Any product that may be evaluated in this article, or claim that may be made by its manufacturer, is not guaranteed or endorsed by the publisher.
